# Invasive Lobular Carcinoma of the Breast With Imaging Features Resembling Mucosa-Associated Lymphoid Tissue Lymphoma: A Case Report

**DOI:** 10.7759/cureus.95440

**Published:** 2025-10-26

**Authors:** Nanae Saito, Yoshika Nagata, Izumi Kinoshita, Toshihiro Saeki, Takahisa Fujikawa

**Affiliations:** 1 Surgery, Kokura Memorial Hospital, Kitakyushu, JPN; 2 Pathology, Kokura Memorial Hospital, Kitakyushu, JPN

**Keywords:** breast, invasive lobular carcinoma, malt lymphoma, surgery, tumor infiltrating lymphocyte

## Abstract

Invasive lobular carcinoma (ILC) is a special histologic type of invasive breast carcinoma characterized by diffuse, single-file infiltration of small discohesive cells. We herein report a case in which differentiation between ILC and primary breast malignant lymphoma was particularly challenging. Imaging findings and core needle biopsy suggested difficulty in distinguishing ILC from mucosa-associated lymphoid tissue (MALT) lymphoma, mainly because the lesion demonstrated dense lymphocytic infiltration. Excisional biopsy ultimately confirmed the diagnosis of ILC. This case highlights the diagnostic challenge posed by ILC presenting with atypical imaging features and marked lymphoid infiltration, which can closely mimic lymphoma. Given the substantial differences in treatment strategies between ILC and primary breast lymphoma, accurate diagnosis necessitates careful integration and summation of imaging, pathological, and immunohistochemical data.

## Introduction

Invasive lobular carcinoma (ILC) accounts for approximately 5% of breast cancer cases in Japan [1] and is classified as a special histologic subtype according to the WHO classification. ILC is characterized by poor cell-to-cell adhesion, which complicates imaging diagnosis, particularly in cases without distinct mass formation. Because of its high frequency of multicentric development and strong invasive tendency, preoperative pathological confirmation is essential for determining the appropriate surgical procedure [2,3]. The prognosis for ILC is generally comparable to that for invasive ductal carcinoma. However, ILC exhibits distinct metastatic patterns and tends to spread to the peritoneum, ovaries, and gastrointestinal tract more frequently. While treatment strategies generally follow standard breast cancer protocols, individualized surgical and systemic management is required due to its diffuse and multicentric nature.

Primary breast mucosa-associated lymphoid tissue (MALT) lymphoma is exceedingly rare, representing only about 0.04% to 0.7% of all breast malignancies [4]. Imaging modalities such as mammography (MG), ultrasonography (US), and magnetic resonance imaging (MRI) generally lack characteristic findings, and the diagnosis is usually established by core needle biopsy or excision biopsy. In contrast to ILC, MALT lymphoma is a low-grade B-cell neoplasm with a favorable prognosis, for which localized radiotherapy or immunochemotherapy, rather than surgery, is the standard treatment. Therefore, accurate distinction between ILC and MALT lymphoma is of great clinical importance, as misdiagnosis could result in inappropriate management (either unnecessary systemic therapy or delayed surgical intervention).

We herein report a case in which malignant lymphoma was initially suspected based on preoperative imaging and core needle biopsy findings, but excision biopsy revealed ILC with marked lymphocytic infiltration.

## Case presentation

A 66-year-old Japanese woman was found to have a high-density mass in her right breast during breast cancer screening and subsequently visited a local hospital. Ultrasound examination revealed an irregular mass in the upper-inner quadrant of the right breast. Breast cancer was suspected based on the imaging findings, and a core needle biopsy was performed. However, the biopsy results made it difficult to differentiate between breast cancer and MALT lymphoma, and the patient was referred to our hospital for further evaluation.

Her medical history included surgery for lumbar spinal canal stenosis, medication for hypertension, and cataracts. She had no family history of hematologic disorders or breast or ovarian cancer. No abnormal findings were observed in the breast skin or nipple. Palpation revealed an approximately 2.5 cm elastic hard mass in the upper-inner quadrant of the right breast. There was no swelling observed in the axillary, supraclavicular lymph nodes on the same side as the mass. No significant lymphadenopathy was detected elsewhere, and no abnormal findings were observed in the contralateral breast. Systemic symptoms such as fever, excessive night sweats, and weight loss, which may be signs of lymphoma, were not diagnosed.

Hematological parameters were within normal limits. The blood counts were within the reference ranges at white blood cell (WBC) 3.7 × 10³ (reference value: 3.0-8.9 × 10³)/μL, hemoglobin 13.2 (11.5.7-15.9) g/dL, and platelets 16.7 × 104 (12.0-39.0 × 104)/μL. No abnormalities were observed in the white blood cell differential. Biochemical tests revealed slight elevations in lactate dehydrogenase (LDH) at 302 (124-222) U/L, blood glucose at 110 (70-109) mg/dL, and hemoglobin A1c (HbA1c), reported according to the National Glycohemoglobin Standardization Program (NGSP) [5] standard at 6.1% (4.6-6.2%). Immunological assays showed the following results: β2-microglobulin (β2MG), 1.4 mg/L (0.9-1.9 mg/L); Immunoglobulin G (IgG), 1276 mg/dL (870-1700 mg/dL); IgA, 376 mg/dL (110-410 mg/dL); and IgM, 34 mg/dL (35-220 mg/dL), with no abnormalities detected. Tumor marker levels were as follows: carcinoembryonic antigen (CEA), 3.6 ng/mL (<5.0 ng/mL); cancer antigen 15-3 (CA15-3), 12.3 U/mL (<31.3 U/mL); and soluble interleukin-2 receptor (sIL-2R), 228 U/mL (121-613 U/mL). Screening for infectious diseases revealed negative results for human immunodeficiency virus (HIV) antibodies, human T-lymphotropic virus type I (HTLV-I) antibodies, hepatitis B surface antigen (HBsAg), and anti-hepatitis C virus (anti-HCV) antibodies. The detailed data are summarized in Table 1.

**Table 1:** Laboratory findings WBC: white blood cell, RBC: red blood cell, LDH: lactate dehydrogenase, HbA1c: hemoglobin A1c, NGSP: National Glycohemoglobin Standardization Program, β2MG: β2-microglobulin, Ig: immunoglobulin, CEA: carcinoembryonic antigen, CA15-3: cancer antigen 15-3, sIL-2R: soluble interleukin-2 receptor, HIV: human immunodeficiency virus, HTLV-I: human T-lymphotropic virus type I, HBs Ag: hepatitis B surface antigen, HCV: hepatitis C virus

Parameter	Result	Reference range
WBC	3.7 × 10^3^/uL	3.0-8.9 × 10^3^/uL
RBC	433 × 10^4^/uL	380-539 × 10^4^/uL
Hemoglobin	13.2 g/dL	11.5.7-15.9 g/dL
Platelet	16.7 × 10^4^/μL	12.0-39.0 × 10^4^/μL
Neutrophils	56.0 %	44-73 %
Lymphocytes	37.0 %	19-47 %
Monocytes	5.0 %	2.0-8.0 %
Eosinophils	1.0 %	1.0-9.0 %
Basophils	1.0 %	0.0-2.0 %
LDH	302 U/L	124-222 U/L
Glucose	110 mg/dL	70-109 mg/dL
HbA1c (NGSP)	6.1 %	4.6-6.2 %
β2MG	1.4 mg/L	0.9–1.9 mg/L
IgG	1276 mg/dL	870–1700 mg/dL
IgA	376 mg/dL	110–410 mg/dL
IgM	4 mg/dL	35–220 mg/dL
CEA	3.6 ng/mL	＜5 ng/mL
CA15-3	12.3 U/mL	＜31.3 U/mL
sIL-2R	228 U/mL	121-613 U/mL
HIV antibody	(-)	(-)
HTLV-I antibody	(-)	(-)
HBs antigen	(-)	(-)
HCV antibody	(-)	(-)

**Figure 1:**
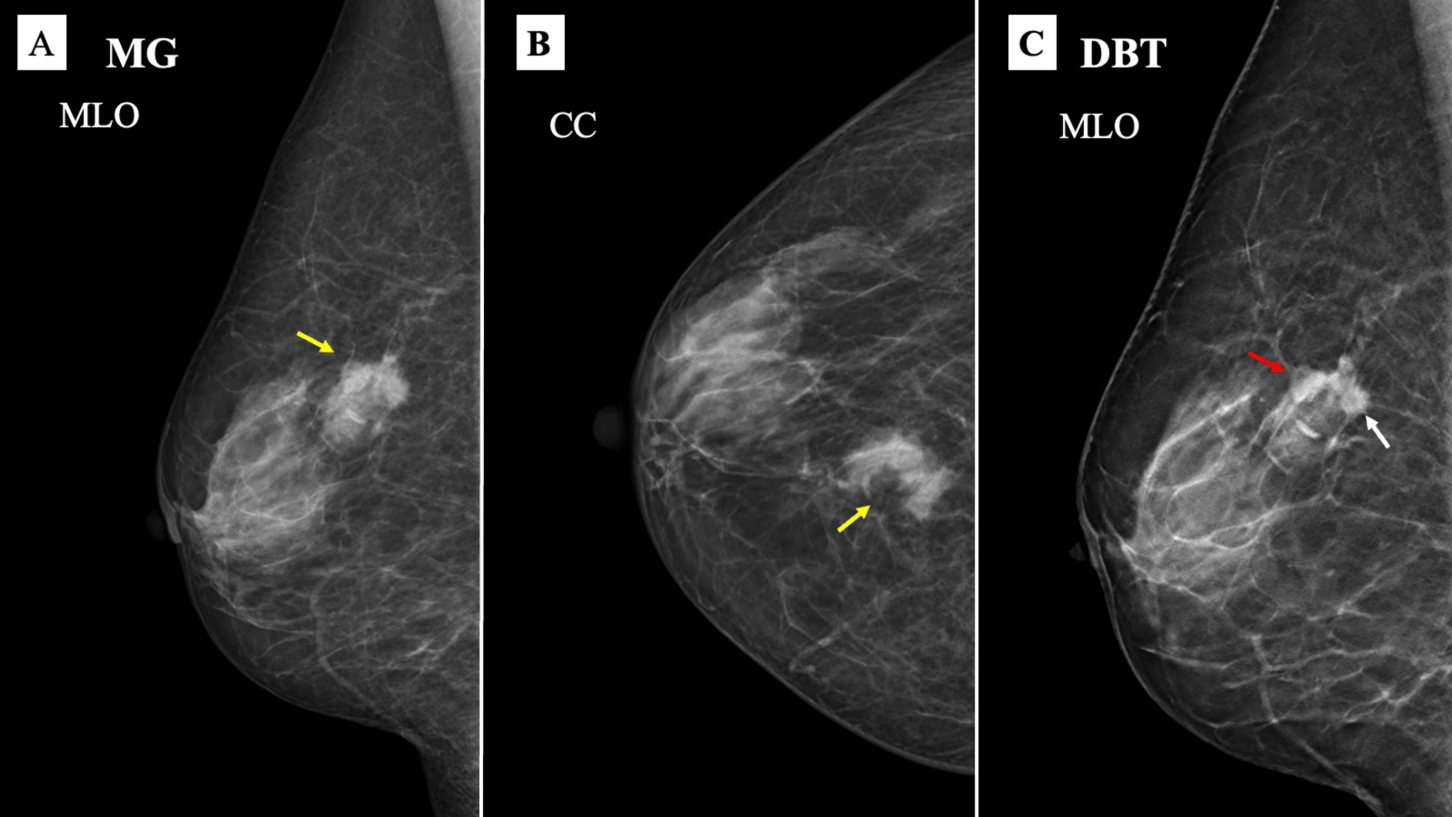
Mammography (MG) findings MG shows a lobulated, high-density mass (yellow arrows) on the medio-lateral oblique (MLO) view (A) and the craniocaudal (CC) view (B). Digital breast tomosynthesis (DBT) (C) revealed a lesion with spiculated margins (white arrow) and smooth, well-defined margins (red arrow). The spicules exhibited imaging characteristics of invasive lobular carcinoma (ILC), while the well-defined areas displayed characteristics of mucosa-associated lymphoid tissue (MALT) lymphoma.

Mammography (MG) of the right breast revealed an irregular, high-density mass with architectural distortion and spiculation, located at the upper to middle border and inner regions, classified as category 4 (Figure 1A-1C).

**Figure 2:**
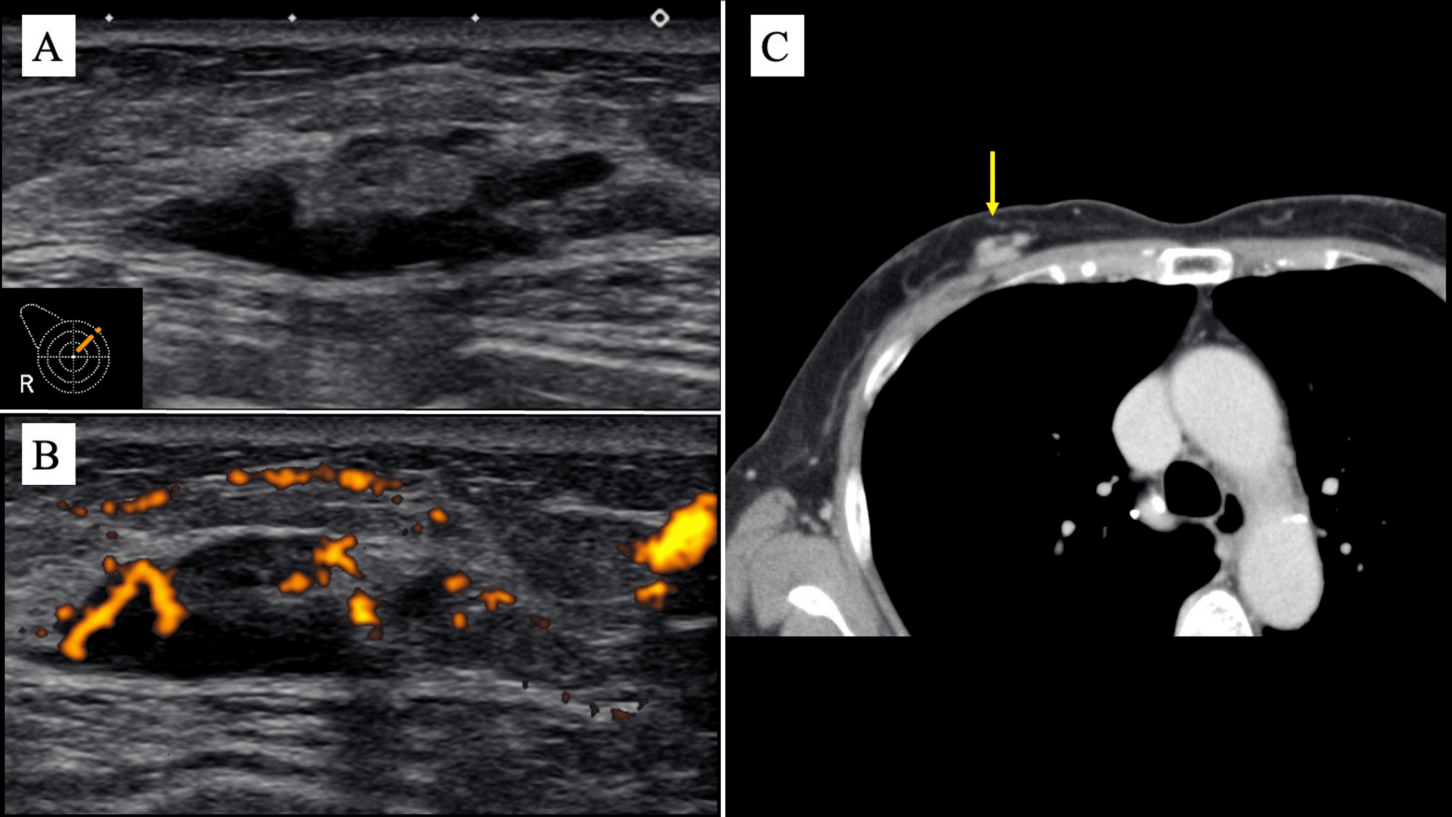
Ultrasonography (US) and contrast-enhanced computed tomography (CT) findings (A) The US demonstrated a mass with relatively uniform and extremely low echogenicity, in contrast to the typical ultrasound image of lobular carcinoma, which shows an irregular, hypoechoic mass. A septal structure was detected within the tumor, and (B) blood flow signals were abundant. (C) CT scans showed a lobulated mass composed of contiguous oval and irregular components, representing a homogeneously enhancing lobulated mass (yellow arrows). These findings are more indicative of malignant lymphoma than of invasive lobular carcinoma (ILC).

The US revealed an irregular, hypoechoic mass measuring 2.7 × 1.6 cm with a septal structure and high blood flow (Figure 2A, 2B). No significant enlargement of the axillary lymph nodes was observed. Contrast-enhanced computed tomography (CT) showed a homogeneously enhancing lobulated mass composed of contiguous oval and irregular components (Figure 2C). No evidence of distant metastasis or lymph node involvement was observed.

**Figure 3:**
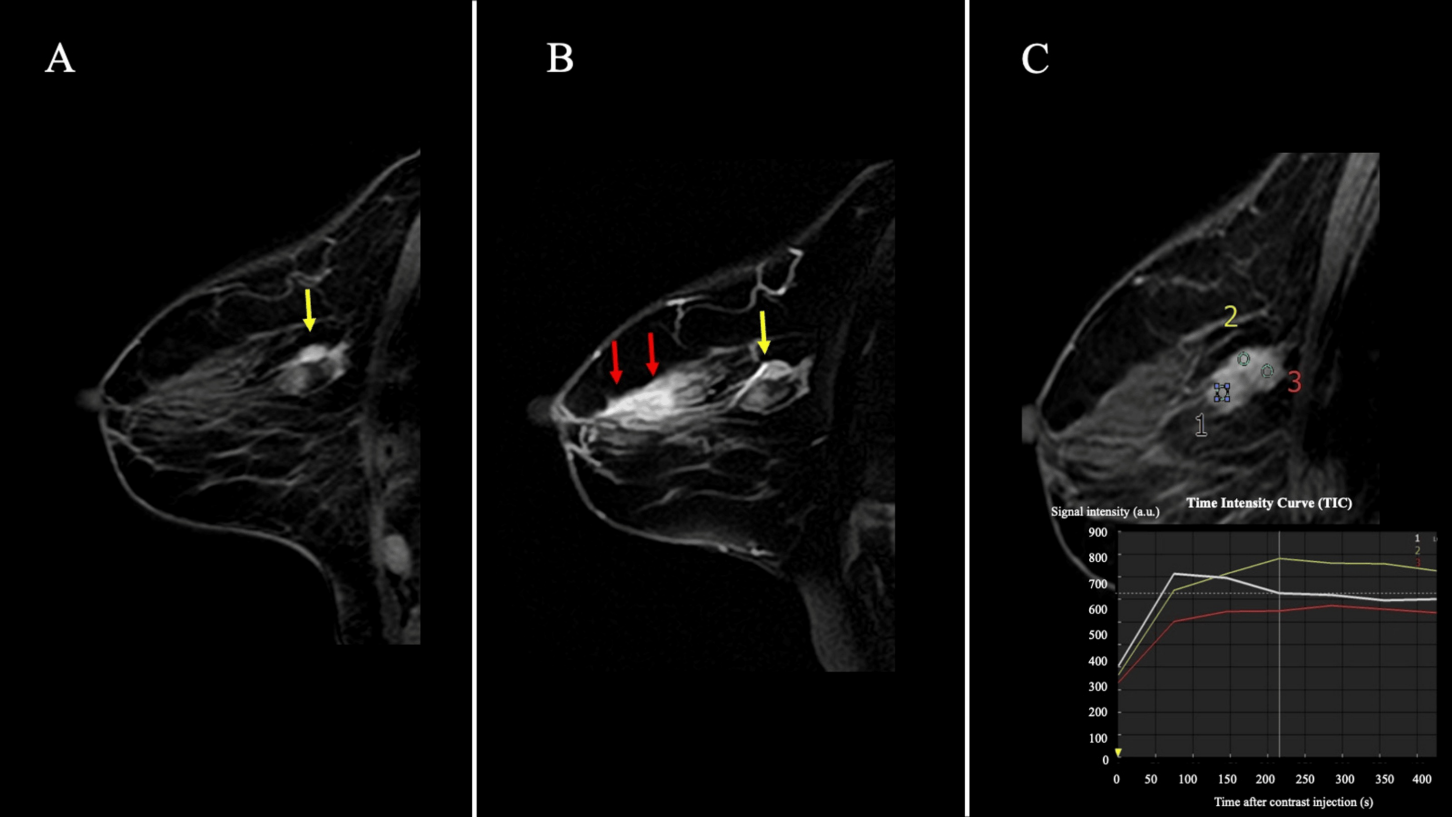
Magnetic resonance imaging (MRI) findings (A) Dynamic early phase on contrast-enhanced fat-suppressed T1-weighted imaging (T1WI). The main lesion is indicated by a yellow arrow. (B) Delayed phase image showing the main tumor (yellow arrow) and intraductal spread toward the nipple (red arrow). (C) Regions of interest (ROIs 1-3) and the corresponding time-intensity curves (TIC). X-axis: Time after contrast injection (s). Y-axis: Signal intensity (arbitrary units, a.u.).

Contrast-enhanced MRI revealed a lobulated mass with irregular margins located deep within the upper inner quadrant of the right breast. On T2-weighted imaging (T2WI), the lesion demonstrated high signal intensity. Diffusion-weighted imaging showed a markedly low apparent diffusion coefficient (mean ADC, 0.84 × 10⁻³ mm²/s). Dynamic contrast-enhanced sequences revealed fast initial enhancement followed by a plateau pattern, with focal areas of washout. Furthermore, irregular peripheral enhancement extending toward the nipple suggested intraductal spread (Figure 3A-3C).

**Figure 4:**
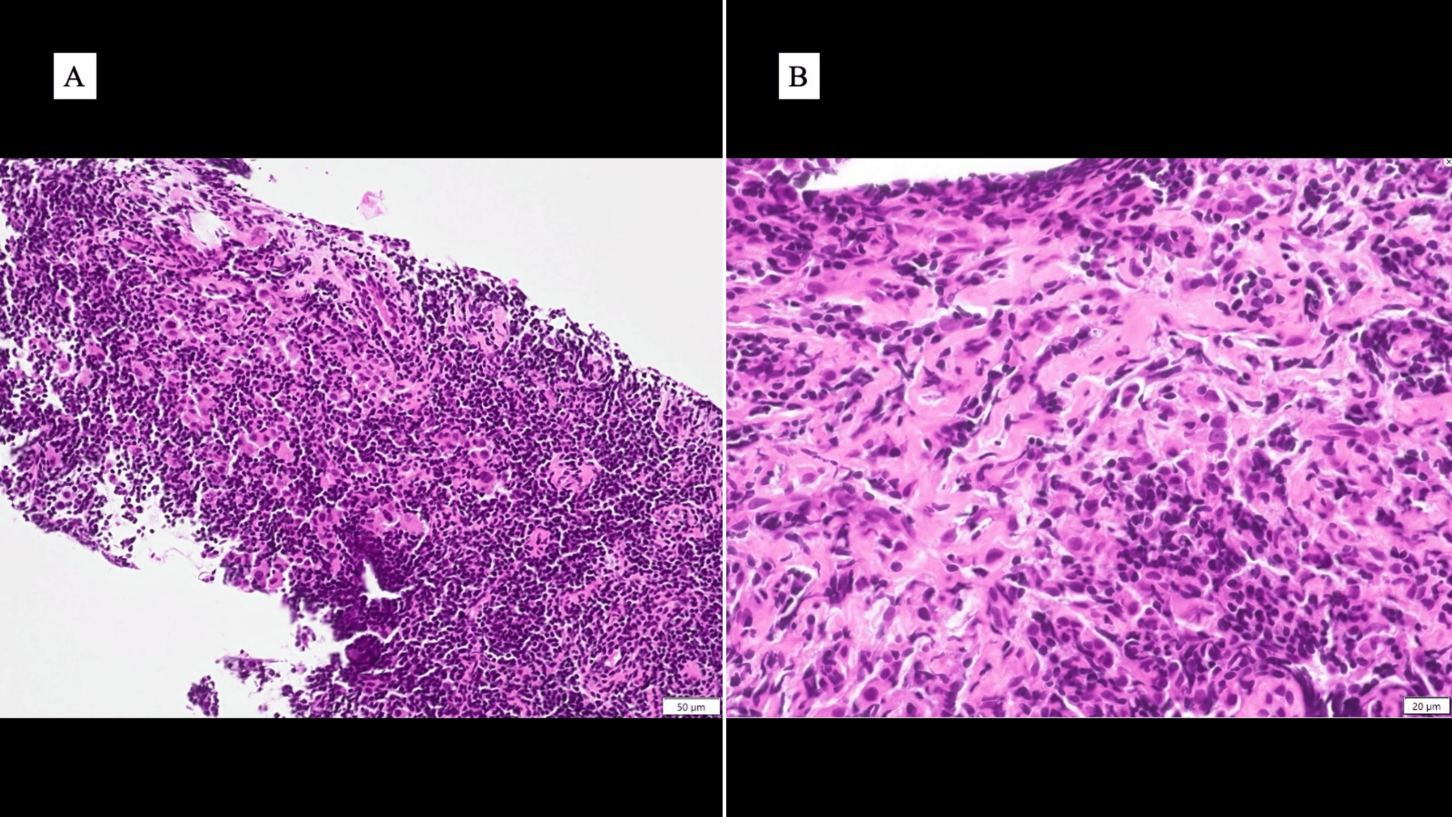
Pathological findings of core needle biopsy (CNB) The fragmented breast tissue obtained by CNB was stained with hematoxylin and eosin (HE), revealing small nests of atypical epithelial cells within a background of dense lymphocytic infiltration. (A) Low magnification (scale bar = 50 μm). (B) High magnification (scale bar = 20 μm).

A core needle biopsy (CNB) performed at the referring institution revealed diffuse infiltration of the mammary gland tissue by small lymphocytes. Scattered clusters of atypical cells were noted among the infiltrating lymphocytes (Figure 4A, 4B).

**Figure 5:**
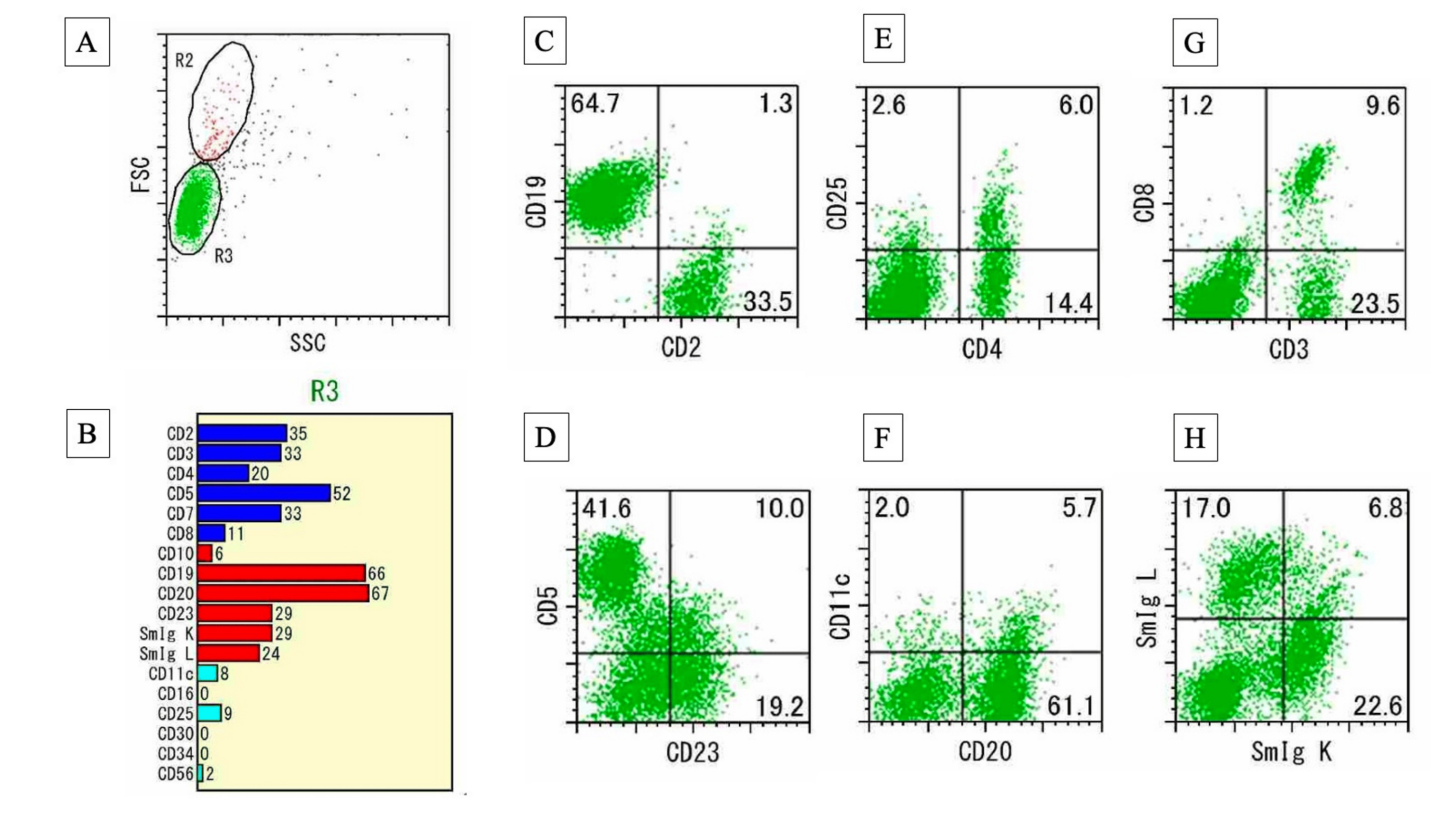
Flow cytometric analysis using the MLA/7-AAD method (A) Gate R3 was defined based on lymphocyte distribution. (B) Event counts for each subset are shown: blue bars indicate T-cell population, red bars indicate B-cell population, and light blue bars indicate other cells. (C) CD19 (vertical axis) vs CD2 (horizontal axis), identifying B-cell and T-cell subsets. (D) CD5 vs CD23 evaluating expression patterns characteristic of B-cell lineage. (E) CD25 vs CD4 showing activated T-cell subsets. (F) CD11c vs CD20 confirming mature B-cell phenotype. (G) CD8 vs CD3 demonstrating cytotoxic T-cell fraction. (H) Kappa vs lambda light-chain expression showing no clear restriction. Cells were analyzed by the MLA/7-AAD method, and nonviable cells were excluded using 7-AAD. FSC: forward scatter, SSC: side scatter, MLA/7-AAD: multi-laser analyzer/7-aminoactinomycin D

Immunohistochemical (IHC) staining demonstrated AE1/AE3 (a pan-cytokeratin antibody cocktail)-positive epithelial cells, whereas myoepithelial cells were not identified. Staining for p40, cytokeratin 14 (CK14), and CK5/6 was negative, supporting the absence of a myoepithelial component. The lymphoid population consisted of a mixture of CD3- and CD5-positive T cells, and CD20-positive B cells. Flow cytometric analysis of the tumor demonstrated a predominance of small B lymphocytes without clear evidence of immunoglobulin light chain restriction (kappa or lambda) (Figure 5).

**Figure 6:**
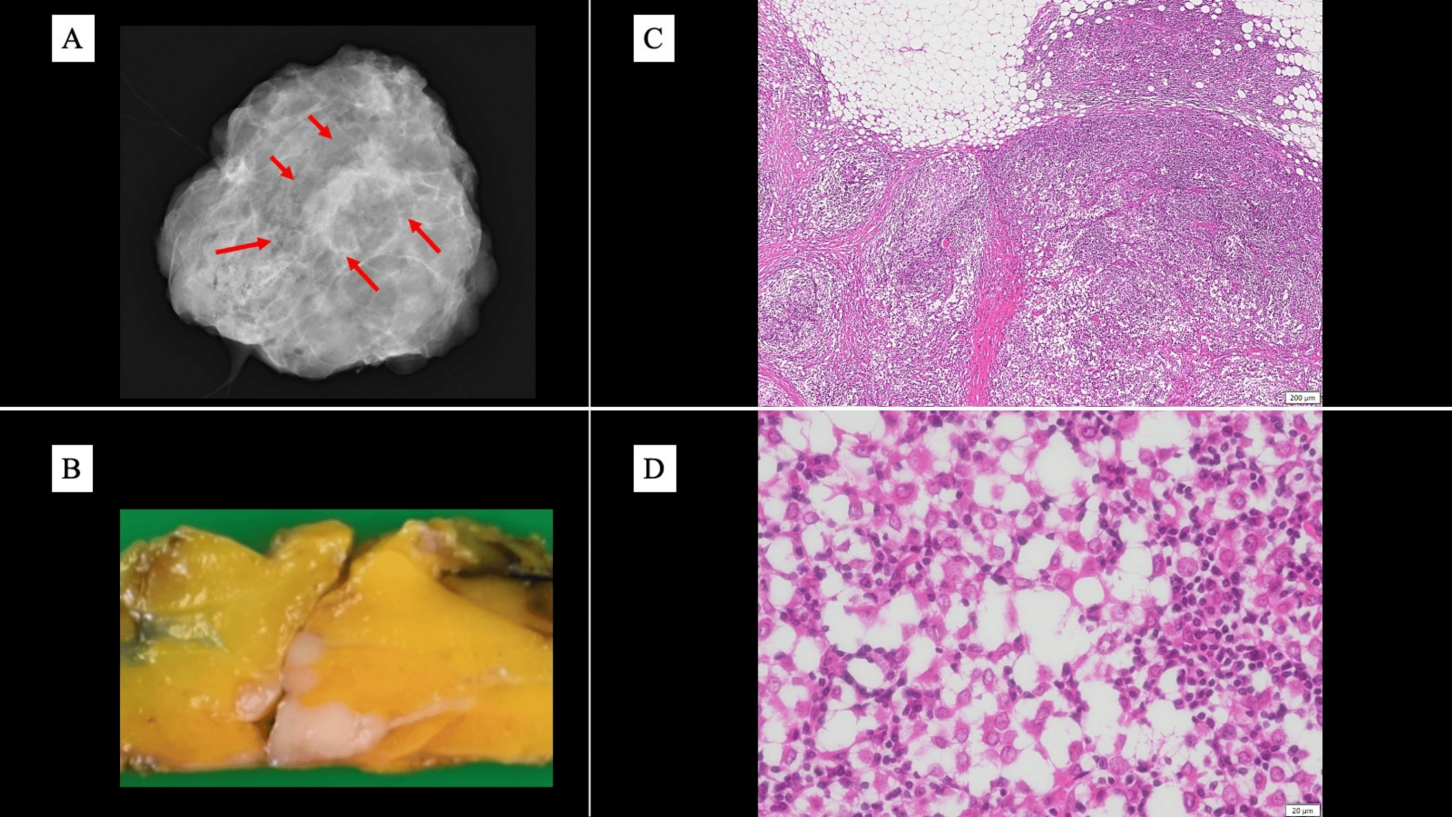
Pathological findings of the surgical specimen The surgical specimen of the breast was stained with hematoxylin and eosin (HE), revealing small nests of atypical epithelial cells within a background of dense lymphocytic infiltration. (A) Photograph of the lumpectomy specimen showing a wide surgical margin. The main lesion is indicated by a red arrow. (B) Gross appearance of the resected tumor showing a whitish mass with relatively well-defined borders. HE staining shows (C) low magnification (scale bar = 200 μm) with alveolar tumor growth and lymphocytic infiltration, and (D) high magnification (scale bar = 20 μm) with atypical epithelial cells.

It was therefore challenging to differentiate among breast cancer with dense lymphocytic infiltration, lymphoid reactive lesions, and low-grade lymphoma. Therefore, an excisional biopsy was performed for definitive diagnosis. A lumpectomy with a wide surgical margin was performed (Figure 6A). Macroscopically, the tumor appeared as a whitish mass with relatively well-defined borders (Figure 6B). Hematoxylin and eosin (HE) staining revealed atypical epithelial cells with pale eosinophilic cytoplasm and oval nuclei, proliferating in an alveolar pattern within a background of dense lymphocytic infiltration (Figure 6C, 6D). 

**Figure 7:**
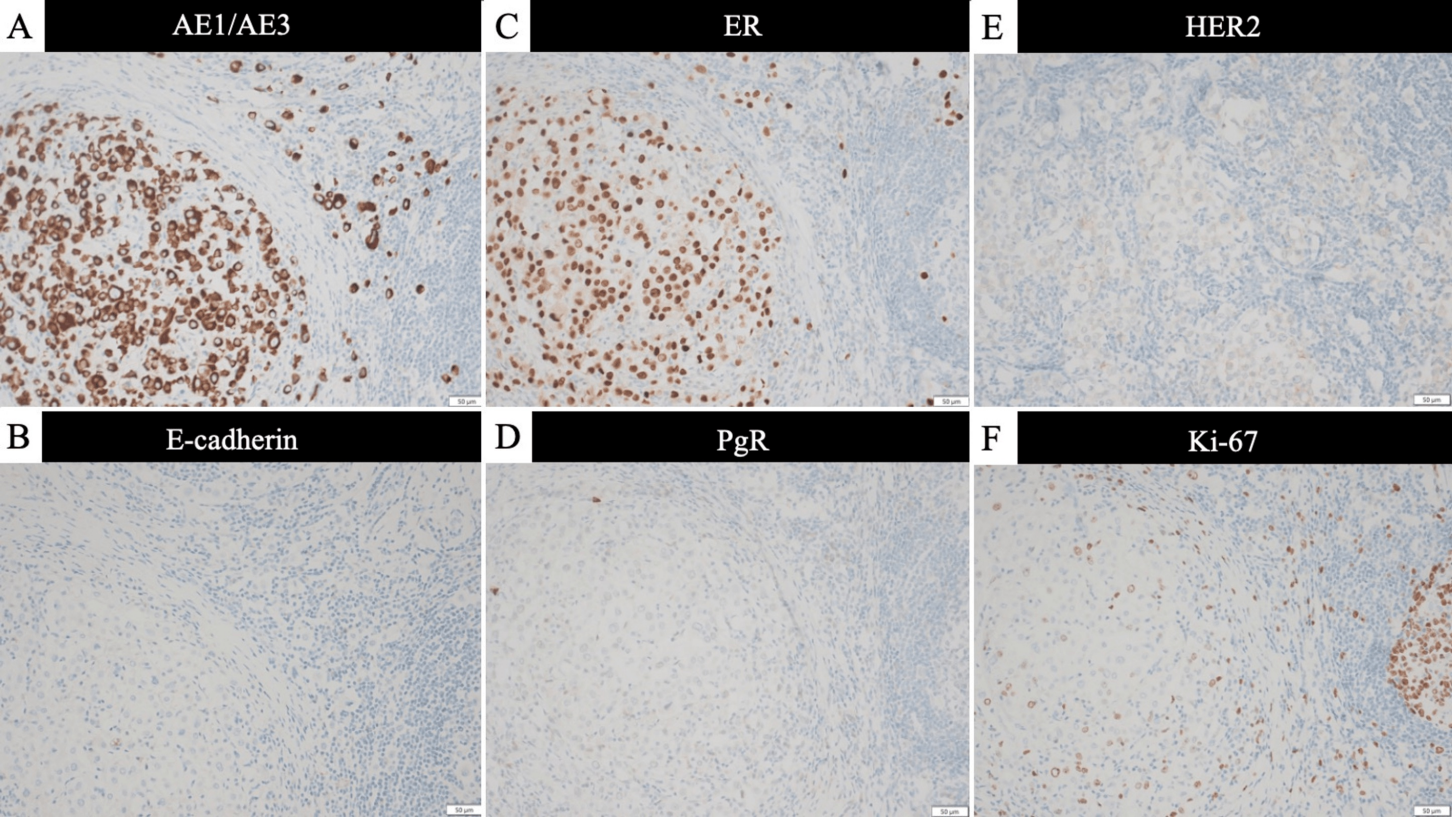
Immunohistochemical findings (A) Tumor cells positive for AE1/AE3. (B) Loss of E-cadherin expression. (C) Strong nuclear positivity for ER. (D) Negative staining for progesterone receptor (PgR). (E) HER2 immunostaining score 1+. (F) Ki-67 labeling index of 35%. (A–F) Representative immunohistochemical staining of the tumor cells at high magnification (scale bar = 50 μm). ER: estrogen receptor, PgR: progesterone receptor, HER2: human epidermal growth factor receptor 2, Ki-67: proliferation marker

Immunohistochemical staining demonstrated positivity for AE1/AE3 (pan-cytokeratin antibody cocktail) and CAM5.2 (low molecular weight cytokeratin antibody), and loss of E-cadherin expression, findings consistent with invasive lobular carcinoma of the breast. Hormone receptor analysis showed that the estrogen receptor (ER) was expressed in more than 95% of tumor cells, the progesterone receptor (PgR) was not expressed (0%), the HER2 score was 1+, and the Ki-67 labeling index was 35%. These findings are consistent with the luminal B molecular subtype (Figure 7A-7F).

Given the diagnosis of invasive cancer, a sentinel lymph node biopsy was performed, which confirmed the absence of axillary lymph node metastasis. As the surgical margins were negative, adjuvant whole-breast irradiation was administered. The final pathological stage, according to the UICC (Union for International Cancer Control) TNM classification (8th edition) [6], was pT2N0M0, stage IIA. The Oncotype DX (21-gene expression assay for breast cancer recurrence risk) test revealed a high recurrence score (RS) of 28, and postoperative adjuvant chemotherapy was administered with epirubicin and cyclophosphamide, followed by docetaxel. The patient subsequently began an aromatase inhibitor as postmenopausal endocrine therapy and remains recurrence-free at 1-year postoperatively.

In reporting this case, we adhered to ethical standards, including the protection of personal privacy, and obtained informed consent from the patient.

## Discussion

Invasive lobular carcinoma (ILC) and primary breast malignant lymphoma may present with overlapping imaging and pathological features, which can complicate diagnosis [2-4]. In particular, ILC may adopt a plasmacytoid morphology that overlaps histologically with the plasma cell-rich component of marginal zone/MALT lymphoma, further hindering accurate distinction. Previous reports indicate that ILC can mimic lymphoma, complicating differentiation between these entities, and have also documented synchronous ILC-lymphoma coexistence within the breast [7-10]. In addition, the differential diagnosis includes neoplastic lesions - mucinous carcinoma; invasive carcinoma of no special type with medullary features; plasmacytoma; and metastatic carcinoma - as well as the inflammatory condition granulomatous mastitis. Because treatment strategies differ substantially, accurate diagnosis is crucial. Our case also presented diagnostic difficulties based on both imaging and pathology, and we discuss the key points of differentiation.

ILC is defined in the WHO classification as a special histologic subtype and is characterized by reduced cell-to-cell adhesion due to loss of E-cadherin, resulting in cord-like or solitary infiltration patterns [11]. While the classical form shows cord-like arrangements, pleomorphic and solid variants have also been described. Imaging findings, particularly on mammography, may include architectural distortion associated with stromal reaction, and when cellular density is high, the tumor may appear as a mass. However, in many cases, a distinct mass is absent. On MRI, a considerable proportion of ILCs present as non-mass enhancement on contrast-enhanced T1-WI, and lesions with low cellularity may demonstrate weak enhancement [12]. Primary breast malignant lymphoma is defined as lymphoma confined to the breast and regional lymph nodes without evidence of systemic disease [13]. It typically presents as a relatively homogeneous mass, and multimodality reviews often demonstrate decreased apparent diffusion coefficient (ADC) values, reflecting increased cellularity and aiding in differential diagnosis [14]. A comparison of images between ILC and MALT lymphoma is summarized in Table 2.

**Table 2:** Comparison of images between ILC and MALT lymphoma ILC: invasive lobular carcinoma, MALT: mucosa-associated lymphoid tissue, MG: mammography, US: ultrasonography, CT: computed tomography, MRI: magnetic resonance imaging

Modality	Feature of ILC	Feature of MALT lymphoma
MG	Irregular, spiculated or ill-defined mass with architectural distortion or focal asymmetric density; rarely calcified.	Oval or round, well-defined mass.
US	Irregular hypoechoic mass with indistinct margins.	Hypoechoic, well-circumscribed mass.
CT	Heterogeneous enhancement.	Homogeneous enhancement with lymphadenopathy.
MRI	Irregular or non-mass enhancement with extensive intraductal component; fast washout.	Well-defined enhancement; plateau or slow washout.

Imaging alone is insufficient for a definitive diagnosis, and histopathological confirmation remains essential. Cytology has limited diagnostic accuracy, and core needle biopsy increases the diagnostic yield. Nevertheless, approximately one-quarter to one-third of cases require excision biopsy for definitive subclassification [15]. In this case, a CNB was conducted at the referring hospital. The region with significant lymphocytic infiltration was probably punctured. A more definitive diagnosis might have been possible if ultrasound imaging had been used to distinguish between the well-defined and the irregularly contoured areas.

Immunohistochemistry is pivotal in differentiation. ILCs are cytokeratin-positive, GATA3 (GATA binding protein 3)-positive, and typically show loss or aberrant expression of E-cadherin, a hallmark that reflects impaired cell-to-cell adhesion. In contrast, lymphomas lack epithelial markers and instead express lineage-specific antigens, such as CD20 in B-cell lymphomas and CD3 in T-cell lymphomas. In addition, light chain restriction (kappa or lambda) and expression of markers such as BCL2 (B-cell lymphoma 2) or MUM1 (multiple myeloma oncogene 1, also known as IRF4) may further support the diagnosis of lymphoma. Recent consensus recommendations emphasize such multiparametric evaluation to standardize interpretation and minimize diagnostic pitfalls [16].

Furthermore, ILC generally demonstrates low tumor-infiltrating lymphocyte (TIL) levels. Large cohort analyses report a median stromal TIL of ~5%, with only a minority showing ≥20% [17]. When present, high lymphocytic infiltration in ILC has been linked to younger age, axillary nodal involvement, higher proliferation, and worse prognosis [18]. In our case, the tumor exhibited unusually high TIL infiltration. This feature contributed to the diagnostic difficulty, as the core needle biopsy (CNB) specimen predominantly sampled lymphocyte-rich areas with only scattered atypical epithelial cells. Consequently, the differential diagnosis included reactive lymphoid lesions and MALT lymphoma. A comparison of the pathological characteristics of ILC and MALT lymphoma is shown in Table 3.

**Table 3:** Comparison of the pathological characteristics between ILC and MALT lymphoma ILC: invasive lobular carcinoma, MALT: mucosa-associated lymphoid tissue, GATA3: GATA binding protein 3, ER: Estrogen receptor, PgR: Progesterone receptor, HER2: Human epidermal growth factor receptor 2

Histopathological category	Feature of ILC	Feature of MALT lymphoma
Cell of origin	Lobular epithelial cells.	B lymphocytes.
Mitotic activity	Generally low grade. High grade for polymorphic forms.	Low grade.
Histology	Loss of cell adhesion, single-file pattern, mild lymphocytic infiltration.	Prominent lymphoid follicles or perifollicular architecture.
Immunohistochemistry	Epithelial cell markers (Cytokeratin and GATA3) positive; E-cadherin negative, ER/PgR/HER2 may also be positive.	Epithelial cell markers negative; and B cell markers (CD20, CD79a, CD19) are positive.

This case highlights the potential for ILC with marked lymphocytic infiltration to mimic primary breast lymphoma, both radiologically and pathologically. Awareness of this overlap is important, as misclassification could lead to inappropriate management strategies. A comprehensive evaluation integrating imaging, pathology, and immunohistochemistry is therefore essential. The accumulation of additional such cases will help refine diagnostic criteria and improve clinical decision-making in these rare but challenging situations.

This report has several limitations. First, it describes a single case, and the findings may not be representative of all ILCs with lymphocytic infiltration. Second, the follow-up period was relatively short, and the long-term clinical outcomes remain unclear. Finally, specimen bias in core needle biopsies may have contributed to diagnostic difficulties.

## Conclusions

In this case, the imaging findings lacked the typical features of ILC, and the tumor was accompanied by marked lymphocytic infiltration, which made pathological distinction from lymphoma difficult. In such situations, it is essential to include both breast cancer and primary breast malignant lymphoma in the differential diagnosis and to comprehensively assess immunohistochemistry, imaging features, and the clinical course. Accumulation of additional cases is expected to further improve the accuracy of differentiation between these two entities.

## References

[1] Adachi Y, Ishiguro J, Kotani H (2016). Comparison of clinical outcomes between luminal invasive ductal carcinoma and luminal invasive lobular carcinoma. BMC Cancer.

[2] Pestalozzi BC, Zahrieh D, Mallon E (2008). Distinct clinical and prognostic features of infiltrating lobular carcinoma of the breast: combined results of 15 International Breast Cancer Study Group clinical trials. J Clin Oncol.

[3] Orvieto E, Maiorano E, Bottiglieri L (2008). Clinicopathologic characteristics of invasive lobular carcinoma of the breast: results of an analysis of 530 cases from a single institution. Cancer.

[4] Shim E, Song SE, Seo BK, Kim YS, Son GS (2013). Lymphoma affecting the breast: a pictorial review of multimodal imaging findings. J Breast Cancer.

[5] (2025). Factors that interfere with HbA1c test results. https://ngsp.org/factors.asp.

[6] Brierley JD, Gospodarowicz MK, Wittekind C (eds) (2017). TNM Classification of Malignant Tumours. https://books.google.co.in/books/about/TNM_Classification_of_Malignant_Tumours.html?id=642GDQAAQBAJ&redir_esc=y.

[7] James ER, Miranda RN, Turner SD (2022). Primary lymphomas of the breast: a review. JPRAS Open.

[8] Kolawole HF, Rai H, Lovrics P, Vasudev P (2024). The alveolar variant of lobular carcinoma and its mimickers: a case series. Cureus.

[9] Anavekar NS, Rozen WM, Rowe K, Murphy C (2008). Synchronous carcinoma and lymphoma of the breast. Clin Breast Cancer.

[10] Hwang C, Krishna A, Kavadi R, Leonard KL (2025). A unique case presentation of synchronous early-stage invasive lobular carcinoma of the breast and marginal zone lymphoma of the ipsilateral breast. Cureus.

[11] Tan PH, Ellis I, Allison K (2020). The 2019 World Health Organization classification of tumours of the breast. Histopathology.

[12] Pereslucha AM, Wenger DM, Morris MF, Aydi ZB (2023). Invasive lobular carcinoma: a review of imaging modalities with special focus on pathology concordance. Healthcare (Basel).

[13] Surov A, Holzhausen HJ, Wienke A (2012). Primary and secondary breast lymphoma: prevalence, clinical signs and radiological features. Br J Radiol.

[14] Jeong S, Kim TH (2022). Diffusion-weighted imaging of breast invasive lobular carcinoma: comparison with invasive carcinoma of no special type using a histogram analysis. Quant Imaging Med Surg.

[15] Frederiksen JK, Sharma M, Casulo C, Burack WR (2015). Systematic review of the effectiveness of fine-needle aspiration and/or core needle biopsy for subclassifying lymphoma. Arch Pathol Lab Med.

[16] De Schepper M, Koorman T, Richard F (2024). Integration of pathological criteria and immunohistochemical evaluation for invasive lobular carcinoma diagnosis: recommendations from the European Lobular Breast Cancer Consortium. Mod Pathol.

[17] Desmedt C, Salgado R, Fornili M (2018). Immune infiltration in invasive lobular breast cancer. J Natl Cancer Inst.

[18] Tille JC, Vieira AF, Saint-Martin C (2020). Tumor-infiltrating lymphocytes are associated with poor prognosis in invasive lobular breast carcinoma. Mod Pathol.

